# Gut microbiota and tumor-associated macrophages: potential in tumor diagnosis and treatment

**DOI:** 10.1080/19490976.2023.2276314

**Published:** 2023-11-09

**Authors:** Dongqin Zhou, Yongsheng Li

**Affiliations:** a The Second Affliated Hospital & Yuying Children's Hospital / The Second School of Medicine, Wenzhou Medical University, Wenzhou, China; b Department of Medical Oncology, Chongqing University Cancer Hospital, Chongqing, China

**Keywords:** Tumor microenvironment, gut microbiota, tumor-associated macrophages, cancer diagnosis, immunotherapy

## Abstract

Avoiding immune destruction and polymorphic microbiomes are two key hallmarks of cancer. The tumor microenvironment (TME) is essential for the development of solid tumors, and the function of tumor-associated macrophages (TAMs) in the TME is closely linked to tumor prognosis. Therefore, research on TAMs could improve the progression and control of certain tumor patients. Additionally, the intestinal flora plays a crucial role in metabolizing substances and maintaining a symbiotic relationship with the host through a complex network of interactions. Recent experimental and clinical studies have suggested a potential link between gut microbiome and TME, particularly in regulating TAMs. Understanding this association could improve the efficacy of tumor immunotherapy. This review highlights the regulatory role of intestinal flora on TAMs, with a focus on gut microbiota and their metabolites. The implications of this association for tumor diagnosis and treatment are also discussed, providing a promising avenue for future clinical treatment strategies.

## 1. Introduction

Tumors pose a significant threat to human life due to the challenges in their early detection and treatment, leading to high mortality rates. The tumor microenvironment (TME) is a complex ecosystem comprising various cell populations and non-cellular components that contribute to tumor growth, invasion, metastasis, and response to therapies.^1,2^ The composition of TME plays a crucial role in the development of solid tumors, making it an essential factor to consider in cancer research. The TME is composed of various immune cells, cancer-associated fibroblasts (CAFs), endothelial cells (ECs), pericytes and other tissue-resident cells.^1,3,4^ These cells and their secreted factors play a crucial role in tumor pathogenesis and progression, offering new avenues for tumor therapy.^3^ Among the infiltrating immune cells, tumor-associated macrophages (TAMs) and their associated inflammatory cytokines are considered to be essential and widespread components of the TME.^5,6^

Macrophages exhibit a remarkable ability to adjust their phenotype in response to varying environmental signals, a process referred to as ‘activation’ or ‘polarization’.^7^ These cells display a high degree of plasticity and are typically categorized into two types: M1, which are classically polarized and exhibit pro-inflammatory properties, and M2, which are alternative activated and possess anti-inflammatory properties. This classification is based on the cells’ phenotype and function. M1 macrophages exhibit a heightened expression of major histocompatibility complex II (MHC-II), which is stimulated by lipopolysaccharide (LPS), interferon gamma (IFN-γ) and tumor necrosis factor alpha (TNF-α). They produce Nitric Oxide (NO), reactive oxygen species (ROS) and reactive nitrogen species (RNS), which have a direct effect on target cells, and also release pro-inflammatory factors like interleukin-1 (IL-1), IL-6 and IL-23 to promote inflammatory responses. These macrophages possess pro-inflammatory and TH1-directed immunostimulatory properties, and are known to function as anti-tumor agents. M2 macrophages can be induced by cytokines such as IL-4 and IL-13. They highly express cluster of differentiation 206 (CD206) and promote helper T cell 2(TH2) cell differentiation by secreting anti-inflammatory cytokines like IL-10, transforming growth factor-beta (TGF-β), arginase-1 (ARG-1) and prostaglandin E_2_ (PGE_2_). These cells play an essential role in tissue remodeling, regeneration, wound healing, revascularisation, anti-inflammation, and promotion of tumor progression.^8,9^ TAMs are involved in key aspects of tumor development, including angiogenesis, invasion, metastasis, and immunosuppression. In the early stages of tumor formation, the majority of macrophages exhibit a classically activated M1 phenotype and perform an anti-tumor role. However, as tumors progress, M1-type macrophages gradually polarize toward M2-type macrophages, which promote tumor progression. Therefore, targeting TAMs for tumor diagnosis and treatment has become a popular research direction in recent years, as enhancing the function of the immune system could be a potential strategy for anti-tumor therapy.

The human gut is home to numerous microorganisms,^10^ collectively known as the ‘gut flora’.^11^ These microorganisms form the second largest symbiotic ecosystem in the body^12^ and have a close relationship with the gut mucosal barrier,^13^ the nutritional status of the body,^14^ metabolic regulation,^15^ and the immune system.^16^ Extensive research into the enteric microbiota has revealed that the intestinal microecology contains a diverse range of bacteria, fungi, viruses, and other eukaryotic microorganisms that contribute to various human diseases to varying degrees.^15^ In recent years, the importance of the enteric microbiota has increased significantly, especially in the detection and treatment of tumors.^17,18^ Recent studies have revealed that microorganisms play a significant role in the TME of certain tumors. The composition of the intestinal microbiota and their metabolites, along with gut homeostasis, have been found to strongly influence the function of immune cells that infiltrate the TME and the evolution of tumor cells.^19^ TAMs, in particular, are a vital component of the TME.^5,20^ Therefore, investigating how the intestinal flora affects TAMs and tumor development may provide a promising avenue for tumor immunotherapy.

This review aims to discuss the impact of gut microbiota on macrophages in the TME and the implications of intestinal flora on TAMs in cancer diagnosis and treatment, highlighting a promising avenue for future clinical treatment strategies.

## 2. Direct effects of gut microbiota on macrophages

### 2.1 Gut microbiota metabolites regulate macrophages

#### 2.1.1 LPS

**Figure 1.**
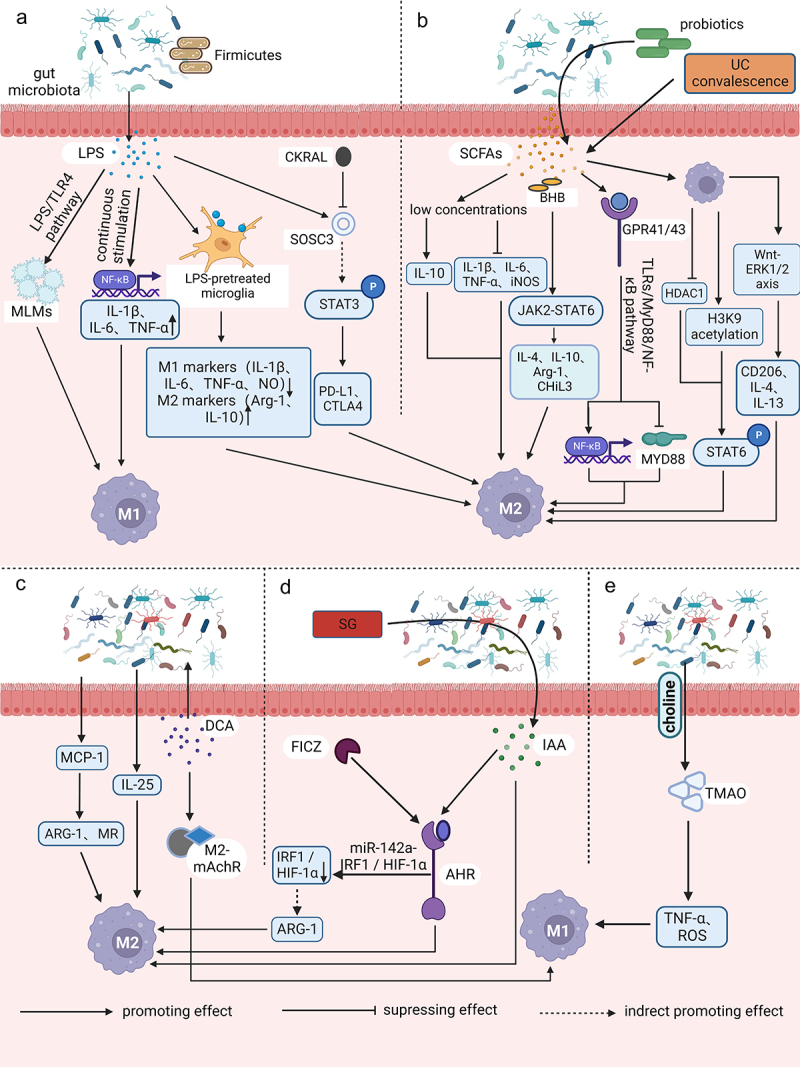
Relationships between gut microbiota metabolites and macrophage polarization. (a) intestinal flora utilizes LPS as a trigger to regulate the aggregation of monocyte-like macrophages through the LPS/TLR4 pathway. This process leads to the formation of a precancerous inflammatory microenvironment that promotes the polarization of M1 macrophages. The process of aging results in the growth of the *Firmicutes* or *Aspergillus phylum* in the intestinal microbiota. This, in turn, leads to an increase in LPS and continuous stimulation of inflammatory signalling pathways in host intestinal macrophages. As a result, pro-inflammatory cytokines TNF, IL-1β and IL-6 are upregulated, NF-κB activation is induced, and M1-like polarization is facilitated. Microglia exhibits an M2-like polarized phenotype as the secretion of pro-inflammatory mediators such as TNF-α, NO, PGE2, IL-1 and 6 is reduced after LPS pretreatment and exposure to LPS stimulation. Additionally, M2 markers such as ARG1 and IL-10 are upregulated. CARKL inhibits the LPS-induced expression of SOCS3, indirectly increasing STAT3 phosphorylation. It also upregulates the expression of immune checkpoint molecules PD-L1 and CTLA-4, and facilitates polarization of M2-like macrophages. (b) low concentrations of SCFAs have been found to suppress pro-inflammatory factors such as TNF-α, IL-1/6 and iNOS, while increasing anti-inflammatory factors like IL-10. This leads to a switch in macrophages towards an M2-like polarization. BHB enhances the expression of M2-related genes, including IL-4/10, ARG1, and CHil3, by strengthening the JAK2-STAT6 signaling pathway, thus directly promotes M2 macrophage polarization. The consumption of complex probiotics has been shown to increase the growth of bacteria that produce SCFAs, specifically butyric and propionic acid. This process also activates the GPR41/43 pathway and the NF-κB cascade response, while inhibiting MyD88 through the TLRs/MyD88/NF-κB signalling pathway. Hence, complex probiotics foster macrophage M2 polarization. Butyrate treatment of macrophages promotes M2 polarization by inhibiting HDAC1 gene expression and enhancing H3K9 acetylation, leading to STAT6 phosphorylation. During recovery from ulcerative colitis, levels of butyrate are greatly increased, which mediates macrophage activation of the WNT-ERK1/2 axis and improves expression of CD206, IL-4, and IL-13, ultimately inducing M2 polarization. (c) DCA induces ecological dysregulation, upregulates MCP-1 expression, and elevates mRNA levels of M2 genes such as ARG-1 and MR, leading to the M2 phenotypic polarization of TAMs. Furthermore, dysbiosis of intestinal flora stimulates IL-25 secretion, which induces polarization of M2 type macrophages. Additionally, DCA increases the mRNA expression level of M2-mAchR in macrophages and promotes M1 macrophage polarization through the TLR2-NF-κB/ERK/JNK pathway. (d) the activation of AhR by IAA is responsible for the M2 TAMs phenotype. SG leads to changes in the intestinal microflora, resulting in an increase in the levels of IAA, which in turn stimulates the polarization of macrophages to the M2 type. FICZ facilitates the expression of AhR, which indirectly leads to an increase in the expression of M2 markers such as ARG-1. This process enhances M2 polarization by downregulating IRF1 and HIF-1α, and inhibiting iNOS expression through the miR-142a-IRF1/HIF-1α pathway. (e) TMAO is created by gut microbes during the breakdown of choline from food. It has been observed to stimulate the production of pro-inflammatory mediators such as TNFα and ROS, which can result in a shift toward the M1 TAM phenotype.

LPS is a component found in the cell wall of Gram-negative (G^−^) bacteria. When these bacteria die, LPS is cleaved and released into the intestinal environment and blood system (Figure 1a). Recent studies have shown a significant association between LPS and TAMs. For example, the pro-inflammatory immune response triggered by microflora plays a crucial role in the development of colon cancer.

During the precancerous stage, the intestinal flora utilizes LPS as a trigger to regulate the accumulation of mononuclear-like macrophages (MLMs) *via* the LPS/toll-like receptor 4 (TLR4) pathway. This results in the formation of a precancerous inflammatory microenvironment, which significantly promotes the polarization of M1-macrophages and the production of pro-tumor inflammatory mediators like IL-1β, IL-6, and TNF-α.^21^ Tan-Garcia *et al*. discovered that activating CD14^+^ human leukocyte antigen DR (HLA-DR)^hi^ CD206^+^ myeloid cells through sequential LPS stimulation resulted in a significant reduction in TNF-α secretion by CD206^−^ myeloid cells in the liver. However, there was no noteworthy reduction in TNF-α secretion by CD206^+^ myeloid cells. The study also found that CD206^+^ myeloid cells in the human liver were not M1-macrophages with pro-inflammatory functions, but instead increased at the end stage of liver disease, leading to a pro-inflammatory microenvironment.^22^ Recent studies have revealed that aging is associated with a rise in the *Firmicutes* or *Aspergillus phylum* to *bacteroidetes phylum* ratio in the intestinal flora.^23,24^ The increase in LPS in the gut microbiota triggers inflammatory signaling pathways in host intestinal macrophages, leading to the upregulation of colonic markers, myeloperoxidase activity, and pro-inflammatory cytokines such as TNF, IL-1β, and IL-6, as well as inducing nuclear factor kappa-B (NF-κB) activation. Consequently, this process causes and accelerates local and systemic inflammation.^23^

Endotoxin tolerance (ET) is a phenomenon in which innate immune cells, such as macrophages/monocytes, exhibit a reduced response to a subsequent endotoxin attack after receiving a prior endotoxin stimulus.^25^ A study found that microglia showed a significant decrease in the secretion of pro-inflammatory mediators like TNF-α, NO, PGE_2_, and IL-1 and 6 when exposed to subsequent LPS after LPS pretreatment. This finding suggests that microglia exhibit a neuroprotective M2-like polarized phenotype.^26^ The development of microglia ET can be regulated by neurons and astrocytes through the release of macrophage colony-stimulating factor (M-CSF). This leads to the activation of its receptor (macrophage colony-stimulating factor receptor, M-CSFR; also known as CSF1R) and downstream extracellular signal-regulated kinase 1/2 (ERK1/2) signaling. Inhibition of M-CSFR activity can reverse the M2 polarizing activity of macrophages/microglia and block glioma progression. The signaling and epigenetic changes conferred by ET seem to be maintained for a longer period in LPS-tolerant macrophages. This means that when these macrophages are exposed to pro- or anti-inflammatory stimuli that induce M1 or M2 polarization phenotypes respectively, they do not return to their original phenotypes.^27^ This indicates that the C-C motif chemokine ligand 2(CCL2), CCL17, and CCL22 are not only markers of M2 macrophages, but are also induced by both LPS and IL-4, which may have a synergistic effect on attracting TH2 cells *in vivo*.^28^The carbohydrate kinase-like protein (CARKL), also called Cingular heptulose kinase, has a direct association with macrophage polarization. It is known to negatively regulate LPS-induced suppressors of cytokine signaling protein 3 (SOCS3), which is a signal transducer and activator of transcription 3 (STAT3) suppressor. This leads to enhanced STAT3 phosphorylation, which in turn drives elevated expression of immune checkpoint molecules such as programmed cell death-ligand 1 (PD-L1) and cytotoxic T lymphocyte-associated antigen-4 (CTLA-4) in the TME. This facilitates the recruitment of immunosuppressive cells like regulatory T cells (Treg cells) and the polarization of M2-like macrophages, ultimately triggering immune escape.^29^

In the organism, the gut and the central nervous system (CNS) are connected through a distinct pathway known as the gut-brain axis.^30,31^ Various bacterial metabolites, including LPS, short-chain fatty acids (SCFAs), serotonin, and dopamine (DA), which are produced in the gut lumen, can influence brain function^32^. This can occur either by stimulating the vagus nerve or by crossing the blood-brain barrier (BBB).^33^ Herbre *et al*. demonstrated that in advanced stages of glioma, the levels of the bacterial metabolite SCFAs were found to be reduced in the cecum of mice. Conversely, the levels of lithocholic acid (LCA) were significantly elevated, providing evidence for the role of LCA in the progression of glioma and its associated lethality.^34^ Additionally, SCFAs were found to regulate the levels of TGF-β and IL-10, contributing to the polarization of microglia toward the M2 phenotype. SCFAs were also shown to inhibit lymphocyte proliferation and T-cell differentiation.^35^ Wang *et al*. demonstrated that the combination of *Bifidobacterium lactis* and *Lactobacillus plantarum* could effectively downregulate the expression of Ki-67 and *N-calmodulin* by inhibiting the phosphatidylinositol 3-kinase/protein kinase B (PI3K/AKT) pathway. Moreover, this combination reduced the abundance of potential pathogenic bacteria such as *Staphylococcus* and *Helicobacter pylori*, while simultaneously upregulating the expression of Occludin in intestinal tissues. These effects ultimately led to a reduction in tumor volume of glioma, an extension of survival time, and the repair of intestinal barrier damage.^36^ Qin *et al*. revealed that DA can influence the shift of TAM toward the M1 phenotype and away from M2 polarization by interacting with dopamine receptor 2 (DR2). Furthermore, they found that the vascular endothelial growth factors/vascular endothelial growth factors receptor 2 (VEGF/VEGFR2) signaling pathway can be used to increase M1 markers such as inducible nitric-oxide synthase (iNOS) and chemokine ligand 9 (CXCL9), while decreasing M2 markers such as CD206 and ARG-1. This reprogramming of M2-polarized macrophages leads to the suppression of glioma growth and normalization of blood vessels. Therefore, targeting tumor microvasculature with DA could be a promising therapeutic strategy.^37^

#### 2.1.2 SCFAs

SCFAs are primarily produced by bacteria belonging to the phylum *Synechococcus* and *Chlamydomonas*. These compounds play a crucial role in regulating dietary fiber and maintaining gut flora homeostasis.^38^ The gut microbiota produces three main SCFAs: acetic acid, propionic acid, and butyric acid. The anti-inflammatory effects of SCFAs are mainly due to their ability to inhibit histone deacetylation (HDAC)^39^ and activating G protein-coupled receptors (GPCR).^38^ Recent studies have shown that human macrophages tend to polarize toward the M2 phenotype when exposed to butyrate and propionate (Figure 1b). For instance, Liu *et al*. found that low concentrations of these SCFAs suppressed the expression of pro-inflammatory factors such as TNF-α, IL-1β/6, and iNOS, while increasing the expression of anti-inflammatory factors like IL-10. This led to M2-like polarization in LPS-stimulated macrophage-like mouse mononuclear macrophages cells (RAW264.7 cells), indicating the anti-inflammatory effects of these SCFAs.^40^ The ketone body β-hydroxybutyrate (BHB) can enhance the expression of genes related to M2 macrophages, including IL-4/10, ARG1, and gibberellin-like protein 3 (CHil3), as well as their downstream effectors. This is achieved by activating the janus kinase 2 (JAK2)-STAT6 signaling pathway, which directly promotes the polarization of M2 macrophages induced by IL-4 and supports the proliferation and mucosal repair of intestinal epithelial cells.^41^ The use of complex probiotics has been shown to promote the growth of bacteria that produce SCFAs, resulting in increased levels of butyric and propionic acid. This process activates G protein-coupled receptor 41/43 (GPR41/43) and inhibits TLR2, 4, 6, and 9-induced myeloid differentiation factor 88 (MyD88), while also activating the NF-κB cascade.^42^ Additionally, complex probiotics down-regulate M1 polarization factors and up-regulate M2 polarization factors, which helps to restore the balance of macrophage phenotype. This ultimately improves mucosal barrier function and reduces inflammation in the colon, potentially making it a useful treatment for colon-associated tumors. In addition, polarization of M2 macrophages is mediated by IL-4-dependent activation of STAT6. Ji *et al*. showed that *ex vivo* butyrate-treated macrophages increased STAT6 phosphorylation by inhibiting HDAC1 gene expression and enhancing Lysine 9 on histone H3 (H3K9) acetylation, resulting in the promotion of M2 polarization and an increase in the expression of M2-associated markers such as ARG1, found in inflammatory zone 1 (Fizz1), and chitinase 3-like 3 (Ym1) in the colon.^43^ Liang *et al*. discovered a decrease in butyrate levels during the active phase and a significant increase during the recovery phase in individuals with ulcerative colitis (UC).^44^ Further investigation revealed that butyrate activated the WNT-ERK1/2 axis and regulated M2 polarization by increasing the expression of CD206, IL-4, IL-13, and other factors. This process helped to rebuild the mucosal barrier and repair damage in the affected patients. The results of the study on zebrafish indicated that butyrate, which is derived from the gut microbiota of zebrafish, reduced the expression of the pro-inflammatory factor TNF-α and increased histone acetylation in zebrafish macrophages. It was observed that this effect was not dependent on GPR81 expression. However, the application of microbially derived propionate to the wound site resulted in a pro-inflammatory effect, with increased aggregation of macrophages and fibrinogen clots.^45^

SCFAs are present in high concentrations in the colon tract, where they are rapidly absorbed, with approximately 90% being absorbed.^46^ The concentration of SCFAs in feces is strongly linked to the development of colorectal cancer (CRC). Butyrate plays a direct role in regulating the apoptosis and proliferation of CRC cells and colorectal stem cells.^47^ In a study conducted by Ma *et al*., it was found that butyric acid and propionic acid negatively regulate HDACs on colonic epithelial cells and immune cells such as macrophages, while also modulating cell-associated transcription factors in CRC. This leads to a reduction in the rate of carcinogenesis.^46^ In a study of mouse intestinal microbiota, researchers discovered that tumor growth led to a significant reduction in the population diversity of the SCFA-secreting phylum *Bacteroides* and *Firmicutes*. However, an increase in the levels of beneficial metabolites, specifically SCFAs, resulted in the attenuation of the PI3K/AKt signaling pathway. This alteration in the pathway led to changes in the expression of certain apoptosis-related genes, ultimately resulting in tumor apoptosis.^46^

#### 2.1.3 Secondary bile acids

Bile acids are synthesized in the liver through the oxidation of cholesterol by specific enzymes. The primary bile acids produced in the liver are cholic acid (CA) and chenodeoxycholic acid (CDCA). These primary bile acids are further metabolized by the gut microbiota through the conversion of 7α-toluenol to form secondary bile acids like deoxycholic acid (DCA) and LCA.^48^ DCA, the primary secondary bile acid, is predominantly synthesized by *Clostridium spp*. of the *Firmicutes* phylum, specifically Gram-positive (G^+^) bacteria such as *Clostridium perfringens* cluster XI and XIVa. This bile acid is commonly associated with the promotion of tumors^49^ and is strongly linked to the development of CRC (Figure 1c). Cao *et al*. found that DCA has the ability to change the diversity of gut microbiota and lead to intestinal ecological dysbiosis. They discovered that DCA is linked to mutations in the adenomatous polyposis coli (Apc) gene during intestinal carcinogenesis. Moreover, DCA increases the presence of opportunistic pathogens such as *Bacteroides fragilis*, *Shigella* and *Desulfovibrio* and decreases the presence of probiotic species such as *Bifidobacterium* and *Lactobacillus* during ecological dysbiosis.^50^

Additionally, the expression of monocyte chemoattractant protein-1 (MCP-1) by tumor cells is linked to the recruitment and regulation of TAMs.^51^ The results also showed that DCA-induced ecological dysregulation increased MCP-1 expression and promoted macrophage infiltration in the intestine. The messenger ribonucleic acid (mRNA) levels of M2 genes, including ARG-1 and mannose receptor (MR), were significantly increased. This led to the recruitment and polarization of M2 phenotype TAMs in the TME. It also induced the activation of Wnt signaling and nuclear β-catenin expression. As a result, the intestinal adenoma-adenocarcinoma sequence was accelerated through the Wnt/β-catenin signaling pathway.^50^ Moreover, Liu *et al*. discovered that mice who had undergone cholecystectomy (removal of the gallbladder) showed an imbalance in their gut microbiota and higher levels of secondary bile acids, specifically DCA and LCA, compared to mice who had not undergone the procedure. The researchers also observed that prolonged exposure to high levels of DCA could contribute to the development of colonic adenocarcinoma from intestinal adenoma.^52^ Intestinal dysbiosis was found to promote the secretion of IL-25, which indirectly contributes to the development of hepatocellular carcinoma by inducing polarization of M2-type macrophages, secretion of CXCL10, and activation of the epithelial-mesenchymal transition (EMT) pathway in hepatocellular carcinoma (HCC).^53^ Additionally, it was found that the TLR4/STAT3 signaling pathway is activated, promoting migration and EMT of human HCC cells.^54^ Wang *et al*. also indicated that the progression of intestinal malignancies may be linked to a decrease in protective SCFAs, an increase in DCA production, and activation of the STAT3 signaling pathway^55^. In a study on intestinal cancer,^49^ it was found that the group of mice administered with DCA showed a significant increase in tumor cells. The expression of small intestinal secretory immunoglobulin A (sIgA) and intestinal immune function was weakened, and the biomarker mouse EGF-like module-containing mucin-like hormone receptor-like 1 (EMR1, also named F4/80) mRNA levels of small intestinal macrophages were increased. As compared to the control group, the expression of MR, ARG-1 and CCL17 were also upregulated, indicating that DCA can promote the polarization of M2 macrophages and in turn lead to tumor cell proliferation and intestinal tumorigenesis. According to some studies, feeding on a high-fat diet may lead to an increase in DCA levels in the fecal and bile pools.^49,56,57^ Ridlon *et al*. have concluded that high fat diet (HFD) could potentially promote colon carcinogenesis by selecting bacteria that metabolize bile acids as toxic secondary bile acids.^57^ According to their proposal, DCA triggers tumor formation by disrupting cell signaling pathways responsible for cell proliferation and apoptosis. This disruption leads to the release of arachidonic acid which is then converted into pro-angiogenic prostaglandins and reactive oxygen species. These species can damage deoxyribonucleic acid (DNA) and inhibit DNA repair enzymes. DCA was found to induce elevation of cyclooxygenase-2 (COX-2) through trans-activation of the epidermal growth factor receptor,^58^ which subsequently led to the proliferation and invasion of colon cancer cells.

The bile acid-activated receptor (BAR) cluster consists of two main receptors: G protein-coupled bile acid receptor 1 (GPBAR1) (also known as TGR5 or M-BAR) and farnesoid X receptor (FXR). GPBAR1 is a receptor for secondary bile acids and is expressed on macrophages.^59^ When stimulated with BAR501 (a GPBAR1 agonist), the expression of M1 phenotype markers such as CD38 and formyl peptide receptor 2 (FPR2) decreased, while the expression of M2 phenotype markers such as IL-10, TGF-β, and c-myc increased in the mouse colon. This demonstrates that GPBAR1 activation effectively promotes the polarization of macrophages to the M2 phenotype.^60^ A previous study discovered that the expression level of FXR mRNA was negatively correlated with the malignancy of human CRC. During the transformation of adenoma to cancer, the expression of FXR was inhibited, and it was not expressed in colon cancer cells. This lack of FXR promoted Wnt signaling, leading to tumor progression in mice.^61^ Yan *et al*. found that the overall FXR signaling deficit resulted in the accumulation of almost all bile acids and increased DCA stimulated macrophages to release TNF-α. This, in turn, may synergistically induce liver injury and enhance hepatotoxicity *via* the FXR-DCA-TNF-α axis with acetaminophen (APAP).^62^ In addition to the typical receptors, DCA stimulation also increased the expression of macrophage M2 muscarinic acetylcholine receptor (M2-mAchR) mRNA in a dose-dependent manner through the TLR2-NF-κB/ERK/c-Jun N-terminal protein kinase (JNK) pathway. This pathway promoted the recruitment and polarization of M1 macrophages and the production of pro-inflammatory cytokines such as iNOS, TNF-α, and IL-6. However, it did not affect the production of M2 macrophage-related genes, which may be regulated by the DCA-M2-mAchR axis.^56^

LCA is known to promote cancer and cause oxidative stress and DNA damage, which in turn promote cell proliferation and tumor development.^63^ Recent research by Shao *et al*. found that LCA inhibits cellular glycolysis, promotes oxidative phosphorylation, and induces macrophages to favor the M2 polarized phenotype while inhibiting their differentiation to the M1 phenotype.^64^ However, these effects are lessened when there is a decrease in the diversity and abundance of the gut microbiota. It is worth noting that Ursodeoxycholic acid (UDCA) is a secondary bile acid present in humans, and intestinal bacteria such as *rumenococci* can form it.^65^ A study conducted by Han *et al*. discovered that tauro-ursodeoxycholic acid (TUDCA) was able to inhibit the phosphorylation of ERK, JNK, and tyrosine phosphoprotein kinase (p38). Additionally, it upregulated CD206 expression which resulted in the induction of M2 polarization of bone marrow-derived macrophages (BMDM)^66^.

#### 2.1.4 Indole acid derivatives

The intestinal flora has the ability to utilize dietary tryptophan and convert around 4–6% of it into major microbial tryptophan metabolites within the intestinal environment.^67^ Several bacterial genera including *Bacillus*, *Bifidobacterium*, *Clostridium*, and *Lactobacillus* have been identified as significant producers of indole.^68^ Additionally, *Lactobacillus royi* has the ability to directly utilize tryptophan to produce several metabolites that are important for immune function.^69^ The aryl hydrocarbon receptor (AhR) is a transcriptional regulator that is responsible for regulating the functions of different immune cells. It can be activated by binding to various ligands, including compounds produced by the dietary metabolism of gut microbes. AhR plays a crucial role in immunity and is considered as an important factor in the immune response.^70,71^ Indoles and their derivatives, specifically indole acid derivatives, are significant ligands of the AhR.^68^ Recent studies have highlighted the crucial role of indoles in regulating the activity, polarization, and growth of TAMs in pancreatic cancer^72^ (Figure 1d). Hezaveh *et al*. demonstrated that indole played a crucial role in determining the immunophenotype of the TME in pancreatic ductal adenocarcinoma (PDAC). The study also revealed that indole-3-acetic acid (IAA) activated AhR, which in turn drove the M2 TAMs phenotype. The absence of AhR resulted in a decrease in TAM proliferation capacity and their extra cellular matrix (ECM) transcriptional signaling, indicating that AhR was a key driver of the pro-tumor phenotype of TAMs. After treatment with the AhR inhibitor CH223191, the survival rate of mice bearing tumors was significantly higher compared to the control group. This suggests that inhibiting AhR could enhance the prognosis of pancreatic cancer and tumors that are present in their original location.^68^ The presence of *L. murinus* in mice was found to result in larger tumors compared to germ-free (GF) mice, and TAMs showed increased expression of pro-tumor genes such as ARG1, indoleamine 2,3-Dioxygenase 1 (Ido1), and IL-10. These findings suggest that *L. murinus* promotes the growth and progression of PDAC, and also triggers an immunosuppressive program in TAMs. The researchers utilized a microbial transplantation technique and found that the presence of indole-producing bacterial microbes in the gastrointestinal environment facilitated tumor growth, enhanced the AhR transcriptional response, and encouraged PDAC immunosuppression of TME formation.

In addition to the major bacterial producers of indole, similar increases in indole production were observed in other bacteria or genera of bacteria known to produce indole, such as *Bacillus copperophilus mimicus*, *Bacillus prussicus fecalis*, *Bacillus lactis* and *Bifidobacterium spp*. These findings suggest that an increase in indole-producing bacteria in the TME is associated with PDAC for tumor resection and overall survival (OS). However, the direct mechanisms by which intestinal flora drive cancer progression remain to be explored.^68^ In addition, Wang *et al*. observed that sleeve gastrostomy (SG) led to alterations in the intestinal flora and elevated levels of IAA, which in turn stimulated the polarization of macrophages to the M2 type. However, the researchers found that only the liver showed an increase in M2 macrophages after SG, suggesting that SG may promote greater infiltration of M2 macrophages.^73^ Yang *et al*. discovered that the compound 6-formylindolyl (3,2-b)carbazole (FICZ) was able to increase the expression of AhR, which in turn led to an increase in the production of ARG-1 and promoted M2 polarization. This was achieved through the downregulation of interferon regulatory factor 1 (IRF1) and hypoxia-inducible factor 1-α (HIF-1α) *via* the microRNA-142a（miR-142a）-IRF1/HIF-1α pathway and the inhibition of iNOS expression.^74^ Wu *et al*. conducted a study to investigate the impact of AHR activation or overexpression on neuroblastoma progression and its potential as a prognostic biomarker. The researchers observed that AHR activation significantly upregulated the expression of kisspeptin 1 （KISS1）, a molecule known to inhibit tumor metastasis. This upregulation, in turn, facilitated the enhancement of kynurenine-mediated neuroblastoma adhesion through the KISS-1- focal adhesion kinase (FAK) axis. Ultimately, these findings suggest that AHR activation may have the potential to curb tumor metastasis in neuroblastoma cases.^75^ Takena *et al*. demonstrated that gliomas can produce kynurenine, which activates STAT2 and STAT1 to enhance AHR expression in TAMs. AHR then recruits TAM through the C-C chemokine receptor 2 (CCR2)/CCL2 pathway, leading to the induction of CD39 and CD73 in TAMs. This synergistically promotes adenosine production, resulting in dysfunction of CD8^+^ tumor infiltrating lymphocyte (TIL) and accelerated tumor progression. Additionally, AHR can inhibit NF-κB signaling *via* the SOCS2- TNF receptor associated factor 6 (TRAF6) pathway and facilitate kruppel-like factor 4 (KLF4)-mediated anti-inflammatory response, ultimately promoting the polarization of TAMs toward M2 phenotype.^76^

#### 2.1.5 Other metabolites

In addition to the common major enteric flora metabolites, there are a few other metabolites that have been reported to regulate macrophages. One such example is the study by Ohue-Kitano *et al*., which found that dietary alanine and its metabolites produced by intestinal lactic acid bacteria promoted M2 macrophage polarization in the presence of IL-4 or IL-13. However, when TH2 cytokines are lacking, adding only fatty acids like alanine does not have much impact. Additionally, the researchers discovered that GPCR40 and its signaling pathway play a role in the M2 polarization process. Conversely, inhibiting GPCR120 leads to a decrease in differentiation.^77^ Likewise, Zhang *et al*. demonstrated that lactate plays a crucial role in inducing TAM differentiation toward M2 (CD206^+^) and has a pro-tumor effect. This effect results in the amplification of tumor invasiveness via the CCL17/CCR4/mammalian target of rapamycin complex 1 (mTORC1) axis.^78^ Additionally, Mirji *et al*. found that trimethylamine N-oxide (TMAO), which is produced by intestinal microbes during the breakdown of dietary choline, can trigger the production of pro-inflammatory mediators like TNFα and ROS. Moreover, TMAO can also reduce the expression of M2-related markers such as IL-10^79^ (Figure 1e). Administration of TMAO or TMA in the TME resulted in polarization of macrophages toward the M1 TAM phenotype, leading to increased survival in PDAC mice. This effect may also be observed in humans. Furthermore, the p38 mitogen-activated protein kinase (P38 MAPK) pathway plays a crucial role in the development of chronic inflammation and cancer. Shi *et al*. found that histamine produced by *Lactobacillus* royale, a microorganism in the gut, can inhibit the development and progression of inflammation-related intestinal tumors. Their research revealed that tumor growth was promoted by activating histamine receptor 1 (H1R) or inhibiting H2R, while histamine effectively suppressed inflammation and colon tumor development by inhibiting LPS-induced TNF and IL-6 expression and p38 MAPK activation in mouse macrophages.^80^ Further exploration is needed to uncover the relationship between metabolites linked to intestinal microbiota and macrophages.

### 2.2 Indirect effects of gut flora on macrophages

**Figure 2.**
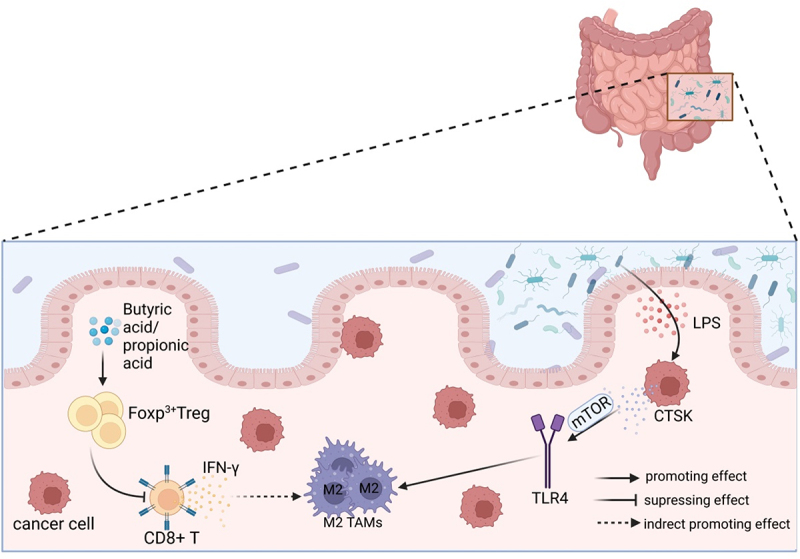
The polarization of TAMs is influenced by gut microbiota through other cells. The metabolites butyric acid and propionic acid produced by intestinal microbes increase the presence of Tregs that express the Foxp3 protein. Tregs indirectly promote the accumulation of M2-like TAMs in the TME by suppressing the secretion of IFN-γ in CD8^+^ T cells. The imbalance of gut microbiota leads to the release of LPS in large quantities. This, in combination with the overexpression of LPS in the TME, results in a significant increase in the secretion of CTSK. CTSK then binds to TLR4 through an mTOR-dependent pathway, which stimulates TAMs to polarize toward the M2 phenotype.

The gut flora can indirectly impact TAMs by acting as an intermediary mediator through other cells (Figure 2).

Studies have demonstrated that a higher abundance of *Bacteroides* and *Bacillus faecalis* in the gut is associated with an increased infiltration of Tregs in the TME of CRC patients.^81^ In mice, the gut microbiota metabolites butyric acid and propionic acid have been found to enhance the production of forkhead box protein P3 (Foxp3)^+^ Tregs.^82^ A study found that CD8^+^ T cells secrete IFN-γ, which has a negative correlation with M2-like TAMs. On the other hand, Treg cells indirectly promote the accumulation of M2-like TAMs by suppressing IFN-γ secretion from CD8^+^ T cells and maintaining the mitochondrial integrity of TAMs in the microenvironment. Additionally, high-grade flow cytometric analysis showed that functional Treg cells can boost the polarization of M2-like TAMs.^83^ M2-like TAMs have a close association with tumor cells that overexpress indoleamine 2,3-dioxygenase (IDO).^76^ The secretion of Kynurenine (Kyn) from IDO or tryptophan 2,3-dioxygenase (TDO) is a core molecular mechanism that regulates the Treg-macrophage suppressor axis and promotes tumor progression through the Kyn-AHR signaling pathway, which involves AHR formation. Gliomas secrete Kyn, which plays a crucial role in promoting tumor progression.^76^ This pathway can affect the activity of M2 TAMs and contribute to the formation of an immunosuppressive TME.^84^ Moreover, Tregs play a crucial role in maintaining free fatty acids (FFA) in TAMs, which is necessary for macrophage survival during M2 polarization. Targeting the lipid metabolism of TAMs through primary fatty acid synthesis could potentially enhance the efficacy of immune checkpoint blockade and serve as a therapeutic tool.^83^

As the primary solid cells in the TME, tumor cells may play a significant role in promoting tumor progression. Recent studies have shown that an imbalance in the gut microbiota can upregulate the expression of histone-degrading enzyme cathepsin K (CTSK), which is secreted by tumor cells and has been linked to tumor metastasis. The researchers discovered that administering Escherichia coli through gavage caused dysbiosis of the intestinal colony, which in turn led to the release of LPS from TME. The binding of LPS to TLR4 was found to contribute significantly to the progression of tumor cells.^85^ In the TME, LPS stimulates the expression of CTSK in collaboration with CRC cells. However, this stimulation does not occur in the absence of CRC cells. Separate administering specific inhibitors of mammalian target of rapamycin (mTOR) and NF-κB pathways, such as rapamycin and BAY11–7082, to block each key nodal pathway showed that tumor-secreted CTSK combined with TLR4 *via* the mTOR-dependent pathway stimulates TAMs to polarize toward M2 phenotype. This, in turn, increases the secretion of cytokines, including IL-10, IL-17, and CD163, and ultimately fosters CRC metastasis and invasive ability through the NF-κB pathway.^86^

The gut microbiota has a direct impact on the synthesis of 5-hydroxytryptamine (5-HT) by colonic enterochromophils in the gastrointestinal tract,^87^ This process is significant in macrophage M2 polarization.^88,89^ Furthermore, 5-HT has been found to increase the proliferation rate and decrease the apoptosis rate of breast cancer cells, which can lead to increased aggressiveness.^90^ Additionally, the receptors 5-hydroxytryptamine receptor 2B (HTR2B) and HTR7 can be considered as macrophage polarization markers, indicating M2 skewed polarization.^89^

## 3. Performance and markers of gut microbiota acting on TAMs in tumor development and diagnosis

### 3.1 The effects of dysbiosis on TAMs in tumor development and its reversal

**Figure 3.**
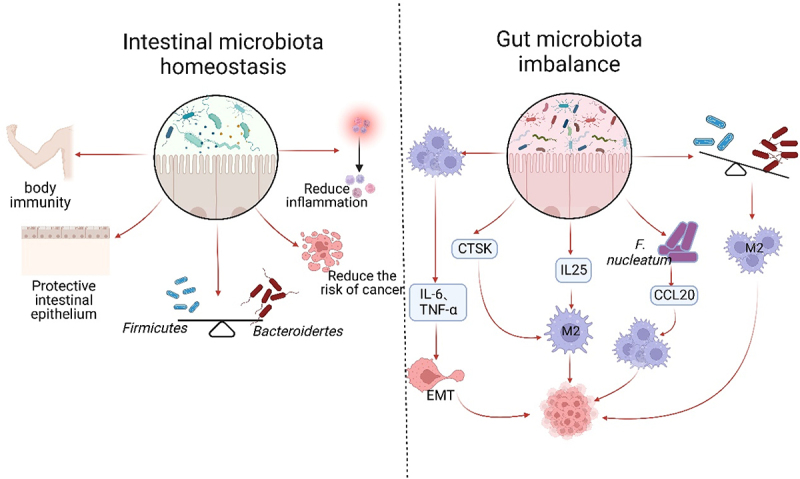
Gut microflora homeostasis and its dysbiosis impact on TAMs. The gut microbiota plays a crucial role in maintaining a balanced immune system and protecting the intestinal epithelium. Under normal conditions, the *Firmicutes* and the *Bacteroides* should be present in equal proportions. This balance helps reduce inflammation, cancer incidence, and supports normal body immunity. However, dysbiosis of the intestinal flora disrupts this balance, leading to the excessive activation of TAMs and the secretion of IL-6 and TNF-α. These factors contribute to tumor invasion, metastasis, and accelerated growth through the promotion of EMT. Dysbiosis also results in increased secretion of CTSK and IL-25, which further promote the polarization of M2 type macrophages and tumor development. The imbalance of flora often leads to an increase in harmful bacteria, such as *F.Nucleatum*. This bacterium can enhance the expression of CCL20, which in turn facilitates the recruitment of TAMs to the TME and promotes tumor growth. Dysbiosis is primarily characterized by a decrease in the proportion of *Firmicutes* and an increase in the phylum of *Bacteroides*. These changes can contribute to the polarization of TAMs into M2 type and expedite tumor growth.

Recent studies have provided evidence that the gut microbiota may aid in the diagnosis of cancer. Specifically, research indicates that individuals with CRC have lower levels of microbial diversity in their gut, which is often linked to an increase in species that promote growth through macrophage-mediated effects, and a decrease in probiotic species like *bifidobacteria* and *lactobacilli*.^91^ The discovery of special AT-rich sequence-binding protein 2 (SATB2) has shown its significance in maintaining intestinal ecological homeostasis and preventing colitis-associated cancer. When SATB2 was deficient in mice, multiple intestinal microflora were disrupted, leading to an increase in both *Bacteroides* and *Bacillus spp*. Additionally, there was an increase in the infiltration of M2 macrophages.^92^ Furthermore, Li *et al*. found that dysbiosis of the intestinal bacterial flora in HCC is enriched with G^−^ bacteria. This can lead to the secretion of IL-25, which triggers polarization of M2 macrophages and accelerates tumor growth and progression.^53^ Zhang *et al*. discovered that there is a correlation between gut microbiota and HCC. They found that after microvascular infiltration of microvascular invasion (MVI) in HCC patients, the gut microbial diversity is reduced. This reduction is indicated by a decrease in the abundance of *Firmicutes*, such as *Bifidobacterium*, and an increase in *Phylum Bacteroidetes*, such as *Klebsiella spp*. and *Prevotella spp*. Additionally, the study found that the elevation of positive infiltration of M2-type TAMs in TME was positively associated with the mTOR signaling pathway.^93^ Interestingly, colonization of mice with *E. coli 541–15* showed a protective effect, leading to reduced levels of TAMs and myeloid-derived suppressor cells (MDSCs), as well as slower adenoma growth in the colonized mice^94^ (Table 1) (Figure 3).

**Table 1.** Presentation and markers of gut microbiota modulating on TAMs in different tumors.

Types of Cancer	Keystone microbiota	The appearance in the tumor	Diagnosis marker	Reference
CRC	*Bifidobacterium* and *Lactobacillus*	Decrease		^91^
CRC	Enteric dysbacteriosis; *Bacteroides* And *Bacillus*	The infiltration rate of M2 macrophages is increased	SATB2	^92^
HCC	Enteric dysbacteriosis	Secretes IL-25, induces M2 macrophage polarization and accelerates tumor growth		^53^
HCC	*Bifidobacterium*; *Klebsiella* and *Prevotella*	*Bifidobacterium* decrease; *Klebsiella* and *Prevotella* increase		^93^
HCC	*Escherichia coli 541–15*	Decrease in TAMs and MDSC levels after colonization, and slowing adenoma growth		^94^
CRC	*Clostridium nucleatum*, *anaerobes*, *P. micra*, *E. cordens* and *X. perforans*	Increase	L-Alanine, Glycine, L-Valine, Myristic acid	^95^
CRC	Enteric dysbacteriosis		CTSK	^86^
CRC	Enteric dysbacteriosis		β-catenin	^96^
Lung Cancer	*Firmicutes* and *Bacteroides SPP*	The lactate content within the tumor is greatly increased, contributing to M2-type polarization	MCT-2	^97^
CRC	*Atopobium vaginae*, *Selenomonas sputgena* and *Faecalibacterium prausnitzii*	Positive association with poor prognosis	*Atopobium vaginae, Selenomonas sputgena* and *Faecalibacterium prausnitzii*	^98^

Previous studies have demonstrated that gliomas can disrupt the gut microbiota in mice, leading to ecological dysregulation.^99,100^ Fan *et al*. found that the composition of the mouse intestinal microbiota changed during glioma growth, with a decrease in S24–7-dominant *anaplastic bacilli* and an increase in *Firmicutes*, such as *Clostridia_Clostridiales, Clostridiales_Lachnospiraceae*, and *Oscillating Spirillum*. This dysregulation of the gut flora can also reduce Foxp3 levels in the brain, worsening glioma progression. Therefore, it may be beneficial to stabilize the intestinal microecology as an adjuvant therapy to slow down glioma progression.^100^ However, it is important to note that studies have shown that a lack of gut microbiota can result in abnormal CNS immune cell function. In GF mice without microbiota, the morphology and gene expression profile of microglia were altered, ultimately promoting glioma progression by increasing the number of immature microglia.^101^

But, the dysregulated gut microbiota’s pro-tumorigenic activity can be altered by macrophage depletion.^91^ Research has shown that intestinal dysbiosis actively contributes to tumor development and EMT. In IDB mice with xenograft tumors, it was observed that the tumors progressed rapidly but the growth rate recovered quickly after macrophage depletion,^102,103^ indicating that the microbiota’s role in tumors primarily depends on the presence of macrophages. Research has shown that an imbalance in the gut microbiome can lead to the overactivation of TAMs, resulting in the secretion of higher levels of IL-6 and TNF-α. This, in turn, inhibits the epithelial marker E-calcineurin and activates the mesenchymal markers N-calcineurin and Vimentin. These changes facilitate the process of EMT and promote the growth and spread of tumors. In contrast, A high-dose antibiotic treatment that leads to macrophage depletion can reverse the findings mentioned earlier,^102^ suggesting that targeted depletion of TAMs and Inflammatory Dendritic Cells (DCs) may become a popular approach in future tumor therapy.^102,103^ Similar results were obtained by Xu *et al*. in an ovarian cancer model, demonstrating that macrophages are aberrantly activated in dysbiosis mice.^103^ Research suggests that depleting macrophages in later stages of tumor growth can effectively prevent further tumor growth. This depletion is accompanied by an increase in the abundance of *Firmicutes* bacterial clades, such as *Lactobacillariidae* and *Clostridiaceae*, which have been found to have antitumor effects. In addition, treatment with clodronate in advanced tumor stages has been shown to effectively reduce M2 phenotypic markers, including IL-10, TGF-β, CCL17, and MCP-1.^104^ *Fusobacterium nucleatum* is a type of bacteria that does not require oxygen to survive and has a rod-like shape. It has been shown to hinder the body’s natural ability to fight against tumors by attracting two types of immune cells known as TAMs and MDSC to the TME.^105^ Xu *et al*. found that high levels of *F*. *nucleatum* in CRC tissues are often accompanied by high levels of CCL20 expression. They also discovered a positive correlation between *F. nucleatum* infection and increased expression of CCL20 and TAMs in the pyrimethamine/dextran sodium sulfate (AOM/DSS) model. Furthermore, *F*. *nucleatum* was shown to promote CRC cell metastasis by regulating the miR-1322/CCL20 axis through the NF-κB pathway and improving the ability to recruit macrophages in TME. Additionally, the expression levels of M2 markers such as CD206, ARG1, IL-10 and TGF-β were found to be upregulated, ultimately promoting CRC tumor progression and metastasis.^106^

### 3.2 Markers for the correlation between gut microbiota and TAMs in the diagnosis of tumors

The use of bacteria, viruses, and fungi as biomarkers for early diagnosis of CRC could be a significant breakthrough in cancer therapy. Understanding their associated pro-tumor behavior may provide valuable insight for effective treatment.^91^ The composition of gut microbiota and metabolites varies throughout the different stages of CRC development. Coker and colleagues found that disorders in the synthesis of aromatic amino acids may contribute to the development of CRC by weakening the intestinal mucosal barrier. They also observed that CRC patients have a higher abundance of certain amino acids, such as L-alanine, glycine, L-valine, and myristic acid, compared to both precancerous (CRA) and healthy (NC) individuals. Notably, the levels of these amino acids progressively increased from normal to CRC stages. There is a growing trend of using bacterial markers such as *Clostridium perfringens*, anaerobic bacteria, *P. micra*, *E. cordens* and *X. perforans* for early detection of CRC. These markers hold potential for serving as promising indicators for the disease.^95^ As mentioned earlier, Intestinal ecological imbalances can regulate the secretion of CTSK by tumors, which in turn activates TAMs in the TME and mediates CRC invasion and metastasis. This positive feedback regulatory system between TAMs and CRC, modulated by CTSK, also identifies CTSK as a valid predictive marker for CRC and a promising therapeutic target and strategy for CRC in the future.^86^ Tikka *et al*. utilized 16S ribosome RNA (16S rRNA) sequencing to determine that arsenic has the ability to cause gut microbial dysbiosis, impair regular immune function, and ultimately trigger the activation of the CRC marker β-catenin.^96^ Moreover, monocarboxylate transporter protein 2 (MCT2) has been strongly associated with various types of tumor tissue, including lung cancer, breast cancer, CRC, and human glioblastoma. Reduced expression of MCT-2 has been linked to tumor progression and aggressive metastatic properties. After specifically knocking out MCT-2, researchers discovered that the intestinal microbiota of *Firmicutes* and *Bacillus spp*. were altered. This led to a significant increase in lactate content in the tumor, which in turn accelerated the acidification of TAMs and prompted their polarization toward the M2 pro-tumor phenotype.^78^ As a result, MCT-2 can be considered a significant biomarker.^97^ Furthermore, Chao *et al*. discovered that malnutrition may have specific modulatory effects on certain microbiota, thereby remodeling the intestinal microenvironment in CRC and accelerating associated tumor progression. *Atopobium vaginae*, *Selenomonas sputigena*, and *Faecalibacterium prausnitzii* may serve as diagnostic markers of malnutrition in CRC patients. These markers were found to be positively associated with a poor tumor prognosis. The treatment of malnourished patients using fecal microbiota transplantation (FMT) revealed that in malnourished patients compared to controls, the levels of IL-10 and CCL1, which are markers for M2, and the M1 marker IL-12 were found to be elevated. To further investigate this, a AOM/DSS mouse model was established. It was observed that FMT from malnourished individuals also resulted in higher levels of IL-10 and CCL1. These elevated levels can contribute to the progression of colon adenoma and the development of chronic colitis into a tumor.^98^

## 4. Conclusion and outlook

Cancer is a worldwide health concern that affects human well-being both physically and mentally. Although chemotherapy and radiotherapy have made significant progress in treating cancer in the past, patients still experience distress. However, in recent years, immunotherapy has emerged as a promising treatment option. By enhancing the patient’s immune system, immunotherapy has improved the quality of life for many patients and increased their survival rate. This approach has revolutionized traditional cancer treatment methods.^107^ Recent research has established a strong correlation between gut microbiota and TAMs in the TME. Several studies have indicated that alterations in the gut microbiota can influence tumor growth and invasion.^97^ For instance, Beame *et al*. found that high doses of specific probiotics, including *Bacillus subtilis*, stimulated macrophage polarization toward M1 and resulted in a significant elevation in CD80 marker levels.^108^ Moreover, several clinical trials have reported a potential association between the gut microbiota and immunotherapy for TME, as shown in Table 2.

**Table 2.** Partial information on clinical trials on gut microbiota and cancer immunotherapy.

Types of Tumors	Staging of tumors	Clinical trial stage	Immunotherapy strategies	reference
Melanoma	Terminal	Stage I	FMT+Navulizumab or Pabolizumab	^109^
Melanoma		Stage I	FMT +Anti-PD-1 monoclonal antibody	^110^
Hematologic malignancies			Chimeric Antigen Receptor T Cell (CAR-T) Therapy	^111^
CRC	Stage Ib/II		Regorafenib + Topalizumab	^112^

This has opened up new avenues for targeted analysis in cancer therapy. Studies have shown that gut microbiota can influence the radiation response of tumors to radiotherapy. For instance, treatment with antifungal antibiotics like fluconazole or 5-fluorocytosine has been found to significantly enhance the growth inhibition of tumors by radiotherapy and improve survival rates in mice with breast cancer and melanoma as compared to antibacterial treatment. In parallel, the tumor suppressive effect of RT may be enhanced by commensal bacteria in the gut, which could lead to a reduction in CD206^+^ F4/80^+^ M2-like TAMs infiltration. Additionally, the study suggests that fungi may be downregulated.^113^ Recent studies have suggested the potential role of gut flora in regulating tumor immunity.^81^ The gut microbiota plays a crucial role in maintaining normal immune system function in the gut and the entire body. A higher concentration of beneficial gut flora can enhance immunotherapy against tumors, thereby inhibiting their progression. Viruses and fungi, alongside bacteria, are important components of the gut microbiota. Chiang *et al*. showed that EBV infection resulted in abundant immune cell infiltration in associated lymphoepithelioma-like cholangiocarcinoma (EBV-LELCC), with an increase in M1 macrophages and a decrease in M2.^114^ While studying hepatitis B virus (HBV) and B-cell lymphoma, Ren *et al*. found that HBV infection was significantly associated with an enhanced risk of follicular lymphoma (FL) and a greater likelihood of further tumor progression. They also showed an increase in M1 macrophage infiltration.^115^Li *et al*. conducted a study where they demonstrated the efficacy of live Neisseria canis in treating B16F10 melanoma. The treatment resulted in the production of TH1-related cytokines in the tumor and spleen, leading to significant tumor cell death and inhibiting the further progression of melanoma. Additionally, the researchers observed an increase in the abundance of probiotics, specifically Lactobacillus, following the treatment.^116^ Shiao *et al*. discovered a significant correlation between decreases in Dectin-1, an essential receptor for detecting fungi, and the prognosis of breast cancer. They found that an excessive presence of Candida albicans in the gut can weaken the effectiveness of radiotherapy and lead to a poorer prognosis. Therefore, enhancing the expression of this receptor could potentially alter the anti-tumor effects following radiotherapy (RT).^113^ Malik *et al*. demonstrated that symbiotic intestinal fungi can hinder the progression of colitis and CRC through the spleen tyrosine kinase (SYK)-Caspase recruitment domain 9 (CARD9) signaling pathway. They found that symbiotic fungi can activate inflammasomes and promote the maturation of IL-18, which in turn prevents colonic inflammation and colon cancer while also mediating anti-tumor T-cell responses. The study revealed that mice lacking CARD9 and SYK had impaired activation of inflammatory vesicles and were more susceptible to colitis and CRC. Additionally, the researchers discovered that depletion of fungi may cause a shift in the overall ecological balance, leading to reduced inflammasome activation and an increased risk of colitis and colon cancer. This suggests that fungal depletion indirectly contributes to the prevention of CRC.^117^ Conversely, an ecological imbalance caused by harmful flora may lead to tumor therapy-resistance or even promote their progression.^118^ Therefore, microbiome-based therapies are a promising area of research in oncology. Inhibiting immune responses associated with microorganisms or their metabolites with precision could be a potential direction for the future.^68^

Gut inflammation is a key characteristic of human obesity, and it can lead to the disruption of the TME by microbes and metabolites. This disruption can accelerate tumor progression and also impact the effectiveness of immunotherapy for specific types of tumors. It was suggested that M2 macrophages were significantly upregulated by obesity only in the presence of tumors.^119,120^ Pingili *et al*. conducted a study to investigate the impact of HFD-induced obesity on the composition of gut bacteria. The results showed that levels of *Enterococcus* and *Lactococcus spp*. were increased, leading to an enhanced abundance of *Clostridium spp*. in the small intestine. However, when anti-programmed cell death protein 1 （PD-1） treatment was administered, there was a notable enrichment of *Bifidobacterium spp*, *Bacillus spp*, *Paenibacillus*, *Cellulosimicrobium*, *Pseudomonas*, and *Comamonas*, as well as *Enterobacteriaceae*. In the obese TME, anti-PD-1 treatment caused a rebound in the intra-tumor M1/M2 ratios. This suggests that the decreased presence of *Anaplasmataceae* and *Enterobacteriaceae*, along with the increased abundance of cecal microorganisms such as *Ruminococcus*, *Lactobacillus*, *Adlercreutzia*, *Mogibacteriaceae*, *Bifidobacterium*, and *Akkermansia*, could potentially serve as representative biomarkers associated with immune checkpoint blockade (ICB). Additionally, in the obesity model, treatment with sodium butyrate (NaB) resulted in a reduction of inflammatory-spectrum immune cell populations, including M1 macrophages and CD8^+^ T cells, while anti-inflammatory cell populations such as M2 macrophages and Tregs cells were increased.^121^ HFD has been found to impact the gut microbiome by reducing the presence of *Clostridium difficile* and increasing the abundance of *Aspergillus* delta and epsilon. Moreover, a study by Li *et al*. demonstrated that leptin stimulates the release of IL-4, IL-8, and IL-10. Elevated levels of leptin can potentially accelerate the progression of obesity-related lung cancer through the activation of the STAT1- solute carrier family 7 member 11 (SLC7A11) pathway, which mediates ferroptosis. Additionally, HFD alters the ratios of *Bacillus* and *Aspergillus* in the gut microbiota, leading to an increase in the CD163/CD206 ratio and promoting M2-type polarization.^122^

On the one hand, intestinal microbiota metabolites play a crucial role in mediating the relationship between intestinal flora and tumor diseases. While the regulatory roles of certain metabolites such as LPS, SCFAs, secondary bile acids, indole, and indole acid derivatives related to M2 type macrophages have been discovered, there are likely other metabolites that regulate the phenotype and functions of TAMs that have yet to be explored. On the other hand, the regulatory chain of the intestinal flora, which indirectly affects TAMs through intermediate agents, requires further investigation. Dysbiosis of the intestinal flora is linked to tumor growth, with an increase in *Bacteroides* phylum abundance and a decrease in *Firmicutes* phylum abundance. It is important to explore therapeutic strategies to restore balance and inhibit tumor growth. This includes investigating how to target and restore the imbalance of the intestinal flora.

Immunotherapeutic strategies targeting TAMs are currently a major area of focus for researchers. TAMs are susceptible to the TME and exhibit pro-tumor effects, according to current consensus.^104^ For cancer therapy, the main mechanisms currently being investigated for TAMs as therapeutic targets are the following^6,123^ inhibition of TAMs production and promotion of their exhaustion^27,91,102,103,124^ modification of the immune microenvironment according to the high plasticity of macrophages, reprogramming TAMs to M1 polarization to control continued tumor growth and enhance the efficacy of immunotherapy^125,126^ inhibition of TAMs recruitment and aggregation.^127^ Therefore, the investigation of gut flora and their metabolites, which can convert pro-tumor M2 TAMs to anti-tumor M1 TAMs, along with identifying biomarkers for tumor diagnosis, shows promising potential. Additionally, studying the negative interactions between gut microbiota, tumor cells, and TAMs, and taking steps to disrupt this harmful connection, could significantly improve the effectiveness of immunotherapy.

## Data Availability

All data are included in the manuscript.
